# Optimization of Synergy Among Granulated Blast Furnace Slag, Magnesium Oxide, and Basalt Fiber for the Solidification of Soft Clay

**DOI:** 10.3390/ma18071577

**Published:** 2025-03-31

**Authors:** Henggang Ji, Xiang Fan, Fan Ding

**Affiliations:** School of Highway, Chang’an University, Xi’an 710064, China; jihenggang@chd.edu.cn (H.J.); dingfan0206@163.com (F.D.)

**Keywords:** waste soft clay, granulated blast furnace slag, central composite design, basalt fiber, reinforcing effect of basalt fiber

## Abstract

In order to reuse granulated blast furnace slag (GBFS) and low-strength soft clay (SC), this study developed a curing material using magnesium oxide (MgO) as an alkali activator to excite the GBFS and basalt fiber (BF) as reinforcing material to prepare the SC. The mixing ranges of GBFS, MgO, and BF were established as 9.48%~14.52%, 0.48%~5.52%, and 0%~1.00454% of the dry clay mass, respectively, and the mixing ratios of the three were optimized using the central composite design (CCD) test. Through the analysis of variance, factor interaction analysis, and parameter optimization of the CCD test, the optimal mass ratio of GBFS, MgO, and BF was determined to be 13.35:4.47:0.26. The curing material of this ratio was named GMBF and mixed with SC to prepare GMBF solidified clay. An equal amount of ordinary Portland cement (OPC) was taken and formed with SC to form OPC solidified clay. The mechanical properties, durability, and hydration products of GMBF solidified clay were clarified by the unconfined compressive strength (UCS) test, freeze–thaw cycle test, X-ray diffraction (XRD) test, and scanning electron microscopy (SEM) test. The UCS of the GMBF solidified clay was 1.08 MPa and 2.85 MPa at 7 and 91 days, respectively, which was 45.9% and 33.8% higher than that of the OPC solidified clay (0.74 MPa and 2.13 MPa) at the same curing time. After ten freeze–thaw cycles, the UCS of GMBF and OPC solidified clay decreased from the initial 2.85 MPa and 2.13 MPa to 1.59 MPa and 0.7 MPa, respectively, with decreases of 44.2% and 67.1%, respectively. By XRD and SEM, the hydration products of GMBF solidified clay were mainly calcium silicate hydrate gel and hydrotalcite. The interface bonding and bridging effect formed between BF and SC or hydration products, indicating that these interactions contributed to the solidified clay enhanced structural integrity. This study demonstrates that the CCD approach provides solution for recycling SC and GBFS. Laboratory tests confirm the potential of the optimized GMBF formulation for practical engineering applications.

## 1. Introduction

With the acceleration of urbanization, the construction of buildings in coastal and lake areas has also increased. The excavated soil from the foundation pit during new construction projects in the areas around the lake is usually classified as waste soft clay [1,2]. This type of soil is often gray in color, has a high moisture content, usually exceeding 40%, and a void ratio generally between 1.0 and 2.0. Due to its characteristics of high moisture content and large void ratio, the mechanical properties of soft clay exhibit significant features of low strength and high compressibility [3]. More than 80% of the waste soil in our country is disposed of by stacking, a method that not only occupies a large amount of land resources but may also lead to environmental issues, such as groundwater pollution or damage to surface vegetation [4]. In this context, the treatment of soft clay is particularly necessary in order to achieve the goal of its reuse.

In both domestic and international contexts, the reuse of soft clay in engineering construction has achieved certain results. For soft clay that has not been contaminated by harmful foreign ions (e.g., lead), it usually only contains organic matter (e.g., humic acid) that affects the properties of the soft clay. When the organic matter content is below 5% [5], prefabricated piles can be made for foundation reinforcement by mixing soft clay with curing agents (e.g., cement, quicklime, ground granulated blast-furnace slag, and silica fume), using stirring equipment [6]. For soft clay with organic matter content higher than 5%, it is necessary to use more effective curing agents (such as calcareous fly ash, silica fume, pozzolanic additives, and NaOH) and mix the curing agents with the clay, using deep mixing equipment to form deep mixing piles, thereby achieving their effective application in engineering construction [7]. These practical applications show that the performance of road subgrade can be effectively enhanced by combining curing agents with the soft clay to provide better engineering characteristics.

Traditionally, ordinary Portland cement (OPC) has been mixed with soft clay to increase the overall strength of the mixture [8,9]. In the process of producing cement, a large amount of greenhouse gas carbon dioxide is emitted, with nearly 900 kg of carbon dioxide emitted for every 1000 kg of cement produced [10]. In 2023, China’s industrial solid waste generation reached 4.28 billion tons, with a comprehensive utilization amount of 2.57 billion tons, resulting in a utilization rate of only 60% [11]. Therefore, it is particularly necessary to conduct research on the resource utilization of waste clay and industrial solid waste. Industrial by-products such as fly ash, silica fume, and granulated blast furnace slag (GBFS) can be used as environmentally friendly materials to replace OPC in soil-stabilization projects [7]. GBFS is a by-product generated during the iron-making process in a blast furnace [12]. Its primary chemical composition includes calcium oxide, silicon dioxide, and aluminum oxide, which closely resemble the oxide composition of OPC.

To improve the utilization of GBFS, research shows that activating GBFS in an alkali environment can effectively enhance its performance as a binding material. This alkali activation not only promotes the hydration reaction of GBFS but also improves its application effects in soil improvement [13,14]. OPC [15,16], alkali slag [17], and lime [18,19] can be used as effective activators for GBFS. These alkaline substances can stimulate the activity of the GBFS, generating gel substances that bond soil particles and provide structural support for the clay, thereby significantly enhancing the overall strength of the clay. Active magnesium oxide (MgO) releases hydroxide ions (OH^−^) during the hydration process to increase the environmental alkalinity, which promotes the hydration reaction of GBFS and enhances its bond performance [20,21]. The production process of cement and lime is accompanied by the emission of carbon dioxide, which has adverse effects on the environment [15,18]. Industrial by-product alkali slag has a high pH value (usually above 12), and the combination of alkali slag with GBFS used to treat clay can lead to an increase in the alkalinity of the clay environment [17]. The pH value of industrial magnesium oxide was usually 10.56, which was the result of laboratory measurements. Therefore, from the perspective of environmental impact, the selection of magnesium oxide as the alkaline exciter of GBFS among OPC, alkali slag, lime, and MgO can effectively reduce the negative impact on the environment. Adding different curing materials to the clay leads to differences in the performance of the solidified clay, which remains to be further explored.

To improve the compressive strength and deformation characteristics of solidified clay, adjusting the amount of curing materials and increasing the reinforcement material is considered an effective method [5]. On the one hand, the curing materials composed of two or more types of materials are used to solidify clay, and their mass usually accounts for 15% of the dry soil mass [22]. An insufficient quantity of curing materials results in low strength of the solidified clay, whereas an excessive amount can lead to a susceptibility to brittle failure [23,24]. On the other hand, adding an appropriate amount of fibers to stabilized soil can effectively improve its brittle failure characteristics and inhibit the further development of shear failure cracks [25,26]. Basalt fiber (BF) is a filamentous mineral mixture made from natural basalt ores at high temperatures. Compared with other synthetic fibers, it has high surface roughness, tensile strength, and deformation modulus, as well as a certain degree of degradation [27]. BF is recognized as a high-performance reinforcement material and has been increasingly utilized in the field of soil reinforcement [28]. However, there is limited research on the use of BF as a reinforcement material, as well as on the application of GBFS and MgO as bonding materials for the solidification of soft clay. If curing materials consisting of GBFS, MgO, and BF are used to stabilize clay, what differences in mechanical properties are exhibited compared to using cement alone as a curing material? To fully utilize the roles of BF, GBFS, and MgO in the curing materials, it is crucial to optimize their quality ratios using scientific methods.

This study proposes the use of GBFS and MgO as curing materials and BF as reinforcement material to jointly solidify soft clay. The experimental design was carried out using the central composite design in response surface methodology, optimizing the mass ratio of GBFS, MgO, and BF, resulting in a curing material named GMBF. The unconfined compressive strength (UCS) test, water stability test, freeze–thaw cycle test, X-ray diffraction (XRD) test, and scanning electron microscopy (SEM) test were performed on GMBF solidified clay. These tests aim to clarify the mechanical properties, durability, and hydration products of GMBF solidified clay. This study optimized the ratio of GBFS, MgO, and BF to provide solutions for treating soft clay and reusing GBFS.

## 2. Experimental Materials and Methods

### 2.1. Experimental Materials

#### 2.1.1. Soft Clay and Curing Materials

**Figure 1:**
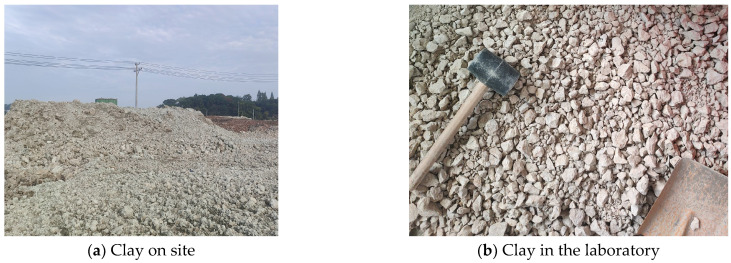
SC used in this study. (**a**) Clay on site; (**b**) Clay in the laboratory.

The soft clay (SC) used in this study was obtained from the excavated soil of a foundation pit in Hangzhou, Zhejiang Province, China, as shown in Figure 1. The basic physical indexes of this clay are shown in Table 1. According to the Chinese Standard for GB/T 50123-2019 standard [29], the natural density (*p*) of the clay was determined by the ring knife method; the plastic limits (*w_P_*) and liquid limit (*w_L_*) of the clay were determined by the liquid and plastic limit water content joint measurement method; the optimum moisture content and maximum dry density were obtained by a heavy compaction test; and the specific gravity (*G_s_*) of the clay was determined using the specific gravity bottle method. The plasticity index (*Ip*) was the difference between the *w_L_* and the *w_P_*, and *Ip* was 19.9. The clay used in this study was defined as low-liquid-limit clay (CL) because the *w_L_* was less than 50% and the *Ip* was greater than 0.73 × (*w_L_* − 20) [30]. The void ratio of clay is calculated by Equation (1). The void ratio of the clay was 1.47, and clay with a void ratio greater than 1 was considered to be highly compressible clay. According to ASTM D2974-14 [31], the loss on ignition (LOI) of the soft clay was measured to be 4.6%, which can be used to characterize the organic matter content.

(1) $$e=\frac{G_{s}p_{w}(1+w)}{p}-1$$

where *e* is the void ratio of clay, *G_s_* is the specific gravity of clay, *p_w_* is the density of water, *w* is the natural moisture content of clay, and *p* is the natural density of clay.

**Figure 2:**
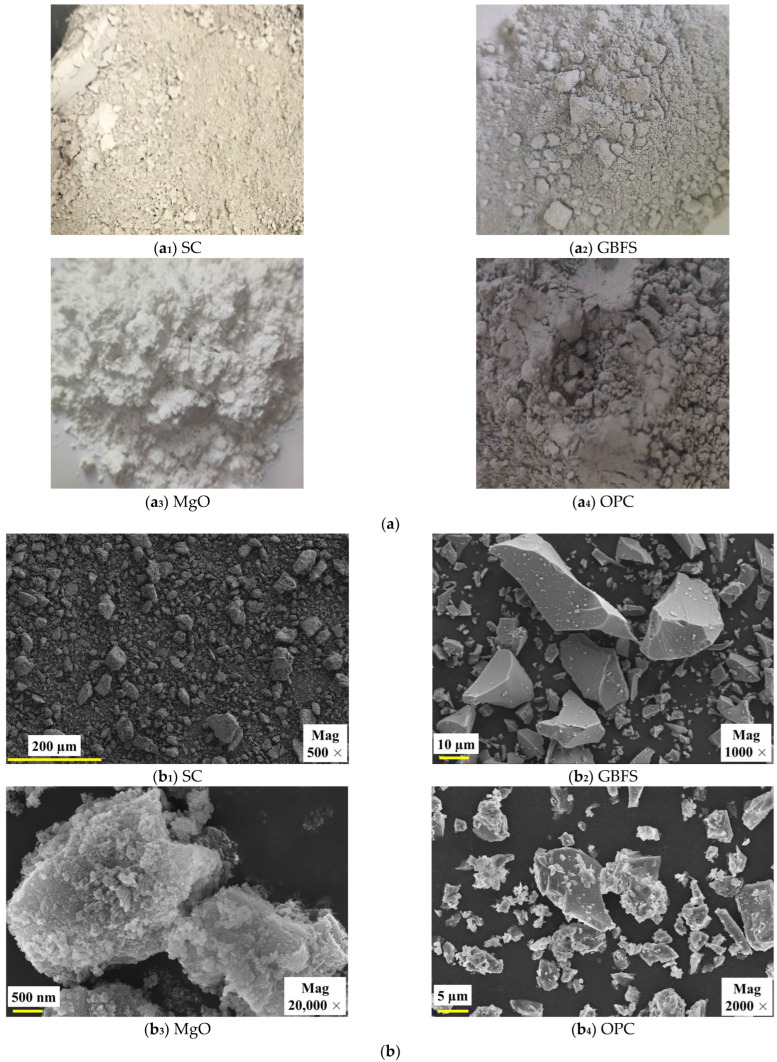
Appearance and microscopic morphology of SC, GBFS, MgO, and OPC. (**a**) Appearance; (**b**) Microscopic morphology.

GBFS and MgO were purchased from Lingshou County Shiyun Mining Products Co., Ltd., which is located in Shijiazhuang City, Hebei Province, China. The 42.5# OPC was purchased from the Building Materials Market, which is located in Xi’an, Shaanxi Province, China. The appearance of SC, GBFS, MgO, and OPC, as well as their microscopic morphology taken by scanning electron microscopy, is shown in Figure 2. As shown in Figure 2a, the appearances of SC, GBFS, MgO, and OPC were light yellow particles, light gray particles, white particles, and dark gray particles, respectively. As illustrated in Figure 2b, SC particles exhibited irregular polygonal shapes, and their surfaces were rough; GBFS particles exhibited an irregular shape yet possessed a smooth surface; MgO particles exhibited an oval shape characterized by an uneven and rough surface; and OPC particles exhibited irregular shapes with rougher surface characteristics. SC, GBFS, MgO, and OPC were present in particulate form, and no linkages were formed between the particles. It is worth noting that the grade of GBFS belongs to S105, which meets the Chinese standard GB/T 18046-2017 [32]. Active MgO is of industrial grade, with a purity of 98%. The OPC meets the requirements of Chinese test standard GB 175-2023 [33], as illustrated in Table 2. The test data indicate that this OPC fully meets national standards, providing reliable data support for its use as a control-group material. The chemical composition and content of the SC, GBFS, MgO, and OPC were measured by an X-ray fluorescence (XRF) spectrometer, as presented in Table 3. The chemical composition of the SC included Al_2_O_3_ and SiO_2_, while GBFS was predominantly composed of CaO, which released Ca^2+^. Additionally, MgO primarily released OH^−^ through hydrolysis (MgO + H_2_O → Mg^2+^ + 2OH^−^). These ions interact in hydration reactions, leading to the formation of gel substances [15,20]. The CaO content in OPC reached as high as 64.55%. The Al_2_O_3_ content of GBFS was significantly higher than that of OPC, while the CaO content of OPC was significantly higher than that of GBFS.

**Figure 3:**
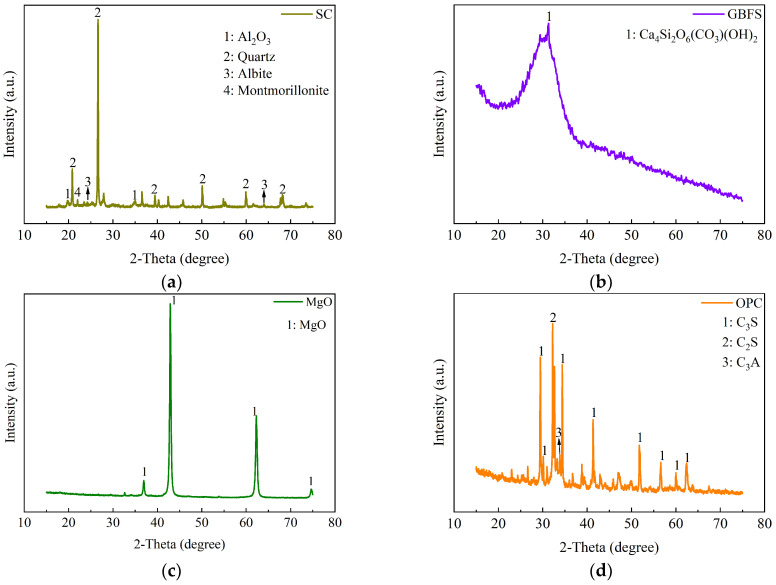
XRD analysis of SC, GBFS, MgO, and OPC. (**a**) SC; (**b**) GBFS; (**c**) MgO; (**d**) OPC.

**Figure 4:**
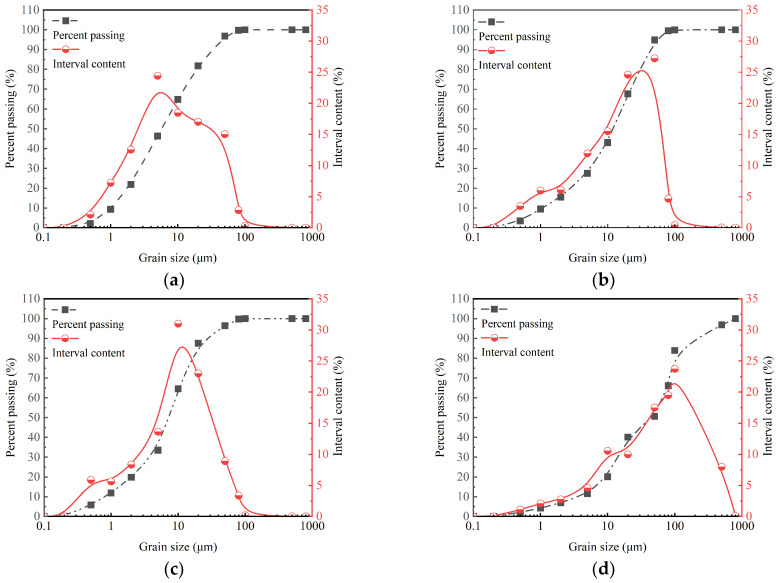
Particle size distribution curves of SC, GBFS, MgO, and OPC. (**a**) SC; (**b**) GBFS; (**c**) MgO; (**d**) OPC.

The mineral compositions of SC, GBFS, MgO, and OPC tested by X-ray diffraction (XRD) are illustrated in Figure 3. The XRD pattern of the GBFS reveals the presence of a complex calcium silicate mineral with the chemical formula Ca_4_Si_2_O_6_(CO_3_)(OH)_2_. The XRD pattern of the clay reveals the presence of SiO_2_, Al_2_O_3_, and montmorillonite. Montmorillonite exhibits the property of expanding in volume when exposed to water. The strong and sharp peak at 2*θ* = 42.9° indicates the high crystallinity of the MgO phase. The XRD pattern of the OPC reveals the presence of C_3_S (3CaO·SiO_2_), C_2_S (2CaO·SiO_2_), and C_3_A (3CaO·Al_2_O_3_). These minerals play a crucial role in the hydration and setting behavior of OPC. The particle size distributions of SC, GBFS, MgO, and OPC were measured with a Bettersize 2000LD laser particle size distribution meter, as shown in Figure 4. In the clay, the content of the fraction of clay (less than 2 µm), silt (2 µm~50 µm), and sand (50 µm~2000 µm) was 21.9%, 74.95%, and 3.14%, respectively [30]. Particle size analysis indicated that 49.35% of the particles in OPC were distributed within the range from 50 to 2000 µm, whereas the particles in GBFS and MgO were primarily concentrated within the range from 2 to 50 µm, accounting for 79.3% and 76.54%, respectively. The smaller particle size not only effectively fills the pores between the clay particles, but its larger specific surface area also significantly promotes the hydration reaction [20,21]. We conducted a particle size analysis on the SC, GBFS, MgO, and OPC to determine the particle size distribution range and the dimensions of the materials utilized in the experiment. The laser particle size distribution meter used in this study is manufactured by Dandong Baite Instrument Co., Ltd., which is located in Dandong City, Dandong, Liaoning Province, China.

#### 2.1.2. Reinforcing Material

**Figure 5:**
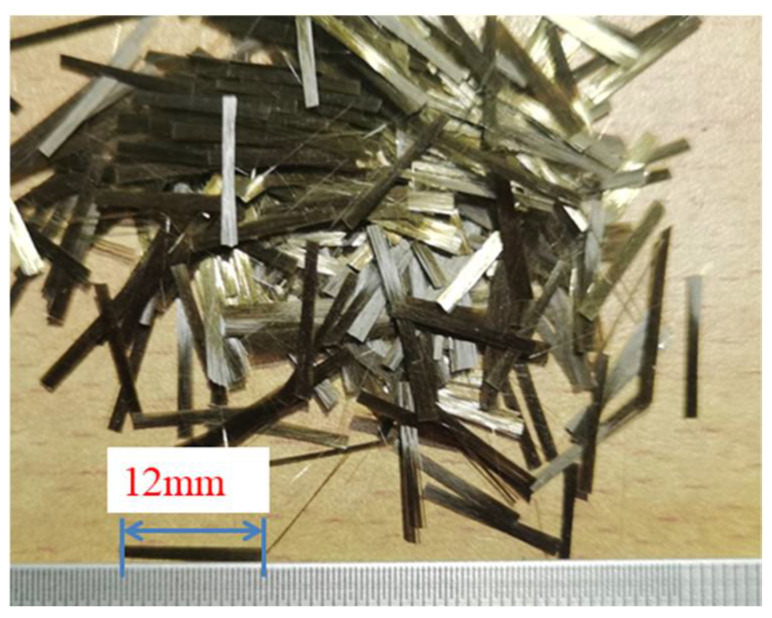
Alkaline-resistant basalt fiber.

Relevant studies indicated that BF was widely utilized in alkaline environments. Some researchers noted that BF demonstrated superior alkali resistance at room temperature compared to glass and carbon fibers [34,35,36]. To mitigate the effects of a strong alkaline environment on the performance of BF, this study selected the alkali-resistant BF produced by Henan Ruison Basalt Fiber Co., Ltd., as shown in Figure 5. The company is located in Zhengzhou, Henan Province, China. The performance parameters of alkali-resistant basalt fiber are detailed in Table 4.

### 2.2. Experimental Design

#### 2.2.1. Preliminarily Determine the Content Range of the Curing Materials

In the alkali-activated slag solidified soil system, active magnesium oxide, carbide slag, and quicklime can be used as effective alkaline activators for the slag, respectively, with a total dosage (slag and activators) typically controlled at 10–20% of the dry soil mass [37,38,39,40]. When these alkaline exciters are used in the appropriate proportion to the slag, the cured soil achieves optimum compressive strength [22,37,38,39,41].

The central composite design (CCD) within response surface methodology (RSM) is utilized to explore the relationship between the mass ratios of GBFS, MgO, and BF and the experimental index of UCS. The CCD requires that the experimental ranges for each factor be predetermined. In the preliminary experiment, the mixing ranges of GBFS, MgO, and BF were established at 9–13.5%, 1.5–6%, and 0–0.8% of the dry clay mass, respectively. It should be noted that the range of selected curing materials is based on the approximate range determined by existing research results, while the range of curing materials more specific and applicable to CCD testing is further determined by actual testing.

#### 2.2.2. Central Composite Design

**Figure 6:**
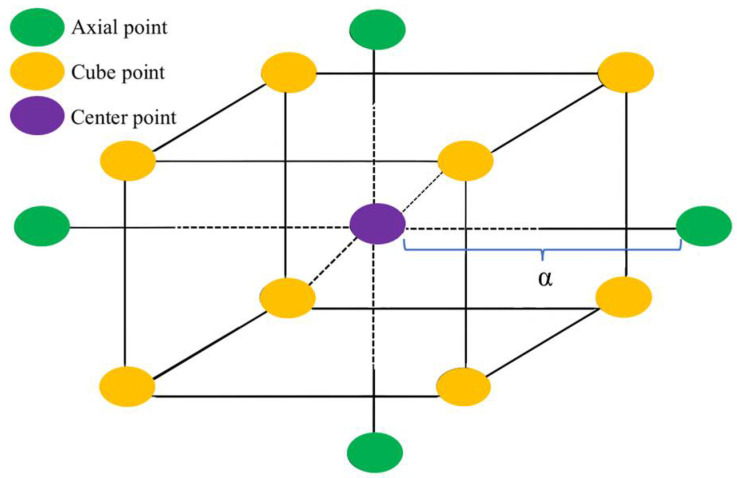
Distribution of test points in central composite design.

Figure 6 illustrates the distribution of test points for the three-factor, five-level CCD. In the preliminary test results of Section 2.2.1, it was determined that the content of GBFS accounts for 10.5~13.5% of the dry clay mass, the content of MgO accounts for 1.5~4.5% of the clay soil mass, and the content of BF accounts for 0.2~0.8% of the dry clay mass. The content range of these three curing materials is suitable for CCD. The encoding conversion can be determined using Equation (2). Based on the obtained design area, a CCD scheme was developed using the response surface design methodology. The independent variables and their coding levels for the rotating center combination design are shown in Table 5. In Table 5, −1 (10.5) corresponds to the coded and actual values of the materials in the curing materials, respectively. The total number of tests for CCD was given by the formula 2*^K^* + 2*K* + *M_o_*, where 2*^K^* represents the cube points, *2K* denotes the axial points, *K* is the number of experimental factors, and *M_o_* indicates the central points. In the equation, the number of tests at the central points was set to six. The independent variables in the experiment included GBFS, MgO, and BF, resulting in a total of three independent variables. Therefore, the total number of tests required was 2*^K^* + 2*K* + *M_o_* = 2^3^ + 2 × 3 + 6 = 20. The central points were represented as zero; the coordinates of the cube points were represented as ±1; and the axial points were represented as ±α, where α was defined as α = 2*^k^*^/4^ = 2^3/4^ = 1.682. In the axial points, *k* represented the number of independent experimental variables [42], which was set to three in this case. According to Equation (2), the CCD experimental ranges for GBFS, MgO, and BF were 9.48%~14.52%, 0.48%~5.52%, and 0%~1.00454% of the dry clay mass, respectively.

(2) $$X_{i}=\frac{x_{i}-x_{0}}{△x_{i}}$$

where *X_i_* is the coded value, *x_i_* is the true value of the impact factor, *x_0_* is the true value of the center level of the impact factor, and Δ*x_i_* is the step size of the change in the impact factor.

A multiple quadratic regression equation (see Equation (3)) is employed to fit the functional relationship between the influencing factors and the response values. The UCS of 7, 28, and 91 days was used as the response value. According to the UCS values of the solidified clay on the 7th, 28th, and 91st days, the optimal mix ratio of the curing materials was determined through variance analysis, model fitting, validation analysis, and parameter optimization.

(3) $$Y=b_{0}+\sum b_{i}X_{i}+\sum b_{ii}{X_{ii}}^{2}+\sum b_{ij}X_{i}X_{j}$$

where *Y* is the response value, *b*_0_ is the intercept, *b_i_* is the linear coefficient, *b_ii_* is the squared coefficient, *b_ij_* is the interaction coefficient, and *X_i_* is the influencing factor.

### 2.3. Sample Preparation

The mixture comprised water, SC, GBFS, MgO, and BF. A critical aspect of preparing this mixture was ensuring the uniform dispersion of BF throughout the soil. If BF bundles were directly mixed and stirred with the curing materials and clay, it could have led to uneven dispersion, resulting in the formation of clumps or spherical structures. In this case, the moisture content of the clay was set to be the same as the natural moisture content (70%). Even if the soft clay had good fluidity, it was still difficult to achieve uniform dispersion of BF. In this context, the preparation process of solidified clay is as follows:

(a) The clay was dried in an oven at 50 °C for 48 h. After completion of drying, it was crushed and passed through a sieve of 2 mm in diameter.

(b) First, the BF was mixed with the dry soil and mixed thoroughly for 5 min using a mixing device. Next, the GBFS and MgO were added and, again, mixed well for 5 min, using a mixing device. Finally, the mass of water required was calculated to be 70% of the mass of dry soil, which was then mixed with SC, GBFS, MgO, and BF, using mixing equipment, for a duration of 5 min. The mixing equipment was a handheld mixer, model BDL-9006. The mixing device used in this study is manufactured by Yongkang City Light Speed Industry and Trade Co., Ltd., which is located in Yongkang City, Zhejiang Province, China.

(c) PVC cylindrical molds with an inner diameter of 39.1 mm and a height of 80 mm were used for the UCS test. The mixture paste was filled into the PVC cylindrical mold in 5 or 6 layers. After adding each layer of the mixture in the mold, a vibrating device was used to vibrate for 5 min to remove bubbles from the sample. Then, the next layer was added until the mold was completely filled. The model number of the electric vibration equipment is HZJ-A. The electric vibration equipment used in this study is manufactured by Beijing Zhongjiao Jianyi Technology Development Co., Ltd., which is located in Beijing, China.

(d) After the mixture paste fills the mold, the top surface of the mold should be covered with plastic wrap and then placed in a curing box for 24 h before demolding. The samples, after demolding, should be sealed in plastic bags and placed in the curing box until they reach the required curing time. In the curing box, the temperature should be controlled at 20 ± 2 °C, and the humidity should be maintained at over 96%.

### 2.4. Testing Method

#### 2.4.1. Test Method for Unconfined Compressive Strength

The test method for UCS was based on the T0805-2024 standard of JTG 3441-2024 [43]. A WDW-100D electronic universal testing machine was utilized, featuring a maximum loading capacity of 20 kN. The WDW-100D electronic universal testing machine used in this study is manufactured by Shenzhen Sunway Technology Co., Ltd., which is located in Shenzhen, Guangdong Province, China. The strain-controlled loading rate was set at 1 mm/min. The equation used to calculate the unconfined compressive strength (*R_c_*) is provided below:

(4) $$R_{c}=P/A=4P/\pi D^{2}$$

where *R_c_* is the unconfined compressive strength (MPa); *A* is the cross-sectional area of the sample (mm^2^); *P* is the maximum pressure when the sample is damaged (N); and *D* is the diameter of the sample (mm), taking the value of 39.1 mm. Each group consisted of three sample replicates, and the final unconfined compressive strength was determined from the average strength of these three samples.

The stress–strain curve represents the relationship between stress (*σ*) and strain (*ε*) of the soil during the loading process. Stress is the internal force per unit area within a material (see Equation (5)). Strain is the relative amount of deformation of a material under stress (see Equation (6)).

(5) σ=F/A

where *σ* is the stress of the sample (MPa), *F* is the applied force on the sample (N), and *A* is the cross-sectional area of the sample (mm^2^).

(6) $$\varepsilon =△L/L_{0}$$

where *ε* is the stress of the sample (dimensionless); △*L* is the difference in height of the sample before and after compression (mm); and *L*_0_ is the original height of the sample (mm), taking the value of 80 mm.

#### 2.4.2. Test Method for Water Stability

The water stability test was conducted following the CJ/T 486-2015 [44]. The solidified clay samples were soaked in a water bath environment at 20 °C for one day, ensuring that the water level remained 2 cm–3 cm above the top of the samples. Upon completion of the water bath, excess water on the surface of the samples was absorbed using a dry cloth, followed by the proceed of the UCS test. Based on the test results, the water stability coefficient of the solidified clay was calculated, defined as the ratio of the UCS of the soaked sample to that of the non-soaked sample (see Equation (7)).

(7) $$K=\frac{Y_{\mathrm{water}}}{Y}$$

where *K* is the water stability coefficient of the solidified clay; *Y_water_* is the soaking treatment of the sample one day before reaching the required curing time, with a soaking time of 24 h, and the unconfined compressive strength of the sample is measured at the end of the soaking (MPa); and *Y* is the unconfined compressive strength of the sample measured after curing to the required time (MPa).

#### 2.4.3. Test Method for Freeze–Thaw Cycle

The freeze–thaw cycle test method was conducted according to the T0858-2009 standard, which was derived from JTG 3441-2024 [43]. Specifically, (I) clay mixed with GMBF to form GMBF solidified clay, clay mixed with OPC to form OPC solidified clay, and solidified clay were subjected to a freeze–thaw cycle test after 91 days of standard curing. (II) During the freeze–thaw cycle, the sample was placed in a device set to −18 °C for 18 h and then transferred to a room at 20 °C to thaw for 6 h, which constitutes one freeze–thaw cycle. (III) The freeze–thaw cycles were conducted for 1, 3, 5, 7, and 10 times. After the freeze–thaw cycles, the mass loss rate and volume were first measured, and the mass loss rate is calculated as shown in Equation (8). (IV) UCS tests were conducted on solidified clay to determine the UCS of the freeze–thaw sample, and then their moisture content was measured.

(8) $$W_{n}=\frac{m_{0}-m_{n}}{m_{0}}$$

where *W_n_* is the mass loss rate of the specimen after *n* freeze–thaw cycles, *m*_0_ is the mass of the sample before freeze–thaw cycles (g), and *m_n_* is the mass of the sample in the *n*-th freeze–thaw cycle (g).

#### 2.4.4. Test Methods for Minerals and Microscopic Morphology

Before analyzing the microscopic morphology and mineral phases, representative fragments from UCS test samples were soaked in anhydrous ethanol for 24 h. After soaking, it was dried at a temperature of 50 °C for 48 h. To investigate the mineralogical composition, the freeze-dried fragments were ground and sieved through a 200-mesh sieve (0.074 mm) prior to scanning for 20 min, using a Rigaku SmartLab SE X-ray diffractometer (XRD) equipped with a Cu Kα source, covering a range from 15° to 75°, with a step size of 0.02°. The Rigaku SmartLab SE X-ray diffractometer used in this study is manufactured by Rigaku Corporation, which is located in Tokyo Metropolis, Japan. By analyzing the diffraction patterns, the diffraction peaks were identified and analyzed using the software Jade 6 to determine the presence of various mineral phases in the sample. The dried fragments were coated with a thin layer of gold and then analyzed using a Sigma 300 instrument to identify the hydrated phases and microscopic morphology. The Sigma 300 instrument used in this study is manufactured by Carl Zeiss AG, which is located in Oberkochen, Germany.

In summary, the CCD test can identify the optimal mass ratio among GBFS, MgO, and BF. The combination of GMBF and clay results in the formation of GMBF solidified clay. An equivalent amount of OPC was used, resulting in the formation of OPC solidified clay when combined with the clay. The comparative test scheme for both is shown in Table 6. The mechanical properties, durability, and microstructure of the solidified clay were investigated using the UCS test, water stability test, XRD test, and SEM test, respectively. In Table 6, the values 7(6), 14(13), 28(27), 60(59), and 91(90) refer to solidified clay that was soaked in water for one day after standard curing for 6, 13, 27, 59, and 90 days, respectively.

## 3. Results

### 3.1. Analysis of Optimal Content for Curing Materials

Table 7 illustrates the relationship between the content of curing materials and the UCS of solidified clay. As indicated in Table 7, the UCS of the S-A1, S-A2, S-A3, and S-A4 groups exhibited a tendency to initially increase and then decrease with increasing MgO content at curing times of 7, 28, and 91 days. When MgO accounted for 3% of the dry clay mass, the UCS of the S-A2 group was greater than that of the other groups. In the S-A1, S-A2, S-A3, and S-A4 groups, the curing materials composed of GBFS and MgO constituted 15% of the dry clay mass. The alkaline environment of the solidified clay improves as the MgO content increases, but the GBFS content decreases accordingly, leading to a reduction in the Al_2_O_3_ and SiO_2_ it provides (see Table 3). In this case, the generation of hydration products is insufficient. The S-A4 group exhibited the lowest UCS when GBFS and MgO constituted 9% and 6% of the dry clay mass, respectively. The variation in UCS indicates that higher MgO content does not consistently enhance the UCS of solidified clay. As the mass percentage of GBFS decreased from 12% to 9%, the UCS of the solidified clay correspondingly decreased. It can be concluded that the hydration products of GBFS are the primary contributors to the strength development of solidified clay.

The UCS of the S-B1, S-B2, S-B3, and S-B4 groups initially increased and then decreased as the content of BF increased. The UCS of the S-B3 group was greater than that of the other groups when MgO and BF accounted for 3% and 0.6% of the dry clay mass, respectively. When the content of basalt fibers was increased from 0.6% to 0.8%, the UCS of the solidified clay decreased instead, indicating that a higher fiber content does not yield better results. Excessive basalt fiber can weaken the spatial network structure formed by the fibers within the soil, as the fibers are prone to clumping, which hinders the bonding between soil particles and hydration products, leading to a decrease in UCS. An appropriate amount of fiber, when mixed with curing materials and clay, forms a solid body characterized by a fiber–gel composite structure through hydration reactions [5]. This composite soil, when subjected to pressure, enables the differential deformation between the fibers and the soil to create a spatial constraint effect on the soil particles, thereby enhancing the UCS of the solidified clay. According to the Chinese standard JTG/T F20-2015 on the requirements of waste stabilized material as the base layer of highway pavement for the UCS [45], the material needs to reach 1.0 MPa after a curing time of 7 days. Notably, the UCS of the S-B3 group exceeded 1 MPa at 7 days, thereby satisfying the strength requirements for highway pavement base layers.

The curing materials content corresponding to the test point with a larger UCS of the nearly solidified clay sample was selected to determine the design area in the CCD. Based on the test results in Table 7, the following conclusions can be drawn: the optimal contents of GBFS, MgO, and BF were 12% (10.5%~13.5% can be selected in the experiment), 3% (1.5%~4.5% can be selected in the experiment), and 0.6% (0.2%~0.8% can be selected in the experiment), respectively.

### 3.2. Results of Central Composite Design Tests

According to the CCD (see Table 5), the UCS test results for the solidified clay after 7, 28, and 91 days are presented in Table 8. Table 8 illustrates that the maximum values of UCS for the solidified clay were found in Group Four, where the contents of GBFS, MgO, and BF were 13.5%, 4.5%, and 0.2%, respectively. The maximum UCS values for the solidified clay at curing times of 7, 28, and 91 days were 1.098 MPa, 2.12 MPa, and 3.01 MPa, respectively. It is noteworthy that the lowest UCS values for the solidified clay were observed in Group One, which had GBFS, MgO, and BF contents of 10.5%, 1.5%, and 0.2%, respectively. The UCS values for Group One were 0.501 MPa, 1.07 MPa, and 1.71 MPa for the same curing times.

### 3.3. Analysis of Variance

The quadratic regression model in Design Expert software was utilized to fit the relationships between curing materials (*X_i_*) and UCS (*Y_i_*) (see Equations (9)–(11)), and the significance of the model was assessed by analysis of variance (ANOVA).

The results of ANOVA and significance tests of the regression model coefficients for solidified clay at 7, 28, and 91 days are presented in Table 9. In Table 9, the significance of the regression model is represented by the *p*-value, where a *p*-value less than 0.05 is considered significant, while a *p*-value greater than 0.05 indicates insignificance [46]. The *p*-value of the quadratic regression model for the UCS of the solidified clay was less than 0.0001 after 7, 28, and 91 days, indicating the high accuracy of the model. The UCS model revealed that the *p*-values for the main terms GBFS (*X*_1_), MgO (*X*_2_), and BF (*X*_3_), as well as the interaction terms GBFS-MgO (*X*_1_*X*_2_) and MgO-BF (*X*_2_*X*_3_) for the 7-day solidified clay, were all less than 0.05. This indicated that GBFS, MgO, BF, GBFS-MgO, and MgO-BF significantly influenced the strength of the solidified clay. Similarly, for the 28-day solidified clay, the UCS model showed that the *p*-values for the main terms *X*_1_ and *X*_2_, along with the interaction terms *X*_1_*X*_2_ and *X*_2_*X*_3_, were also less than 0.05, confirming a significant effect of GBFS, MgO, GBFS-MgO, and MgO-BF on the strength of the solidified clay. For the 91-day solidified clay, the UCS model indicated that the *p*-values for the main terms *X*_1_, *X*_2_, and *X*_3_, and the interaction term GBFS-BF (*X*_1_*X*_3_) were less than 0.05, signifying that GBFS, MgO, BF, and GBFS-BF had a significant impact on the strength of the solidified clay.

As shown in Table 9, the correlation coefficient (R^2^) for all three models was greater than 0.95, and the adjusted correlation coefficient (Adj R^2^) was greater than 0.92, indicating that the model effectively simulated the relationship between the response values and impact factors. The difference between the Adj R^2^ and the predicted correlation coefficient (Pre R^2^) of each model was smaller than 0.2, indicating that the model exhibits high fitting accuracy, making the regression equation more reliable. The coefficient of variation (C.V.) of the model was 4.46%, 4.5%, and 4.57% after 7, 28, and 91 days, respectively, all of which were below 5%. The adequate precision (Adeq precision) values of the model were 23.473, 20.79, and 17.26 after 7, 28, and 91 days, respectively, all of which exceeded 4 [6]. This indicates that the model can effectively guide the design.

(9) $$Y_{7d}ucs=0.95+0.11X_{1}+0.11X_{2}+0.051X_{3}+0.03X_{1}X_{2}-0.082X_{2}X_{3}-0.077{X_{1}}^{2}-0.047{X_{2}}^{2}-0.043{X_{3}}^{2}$$

(10) $$Y_{28d}ucs=1.78+0.21X_{1}+0.16X_{2}+0.088X_{1}X_{2}-0.13X_{2}X_{3}-0.12{X_{1}}^{2}-0.08{X_{2}}^{2}-0.063{X_{3}}^{2}$$

(11) $$Y_{91d}ucs=2.8+0.26X_{1}+0.23X_{2}+0.14X_{3}-0.16X_{2}X_{3}-0.2{X_{1}}^{2}-0.13{X_{2}}^{2}-0.12{X_{3}}^{2}$$

### 3.4. Graphical Interpretation and Analysis of Factor Interaction Effects

**Figure 7:**
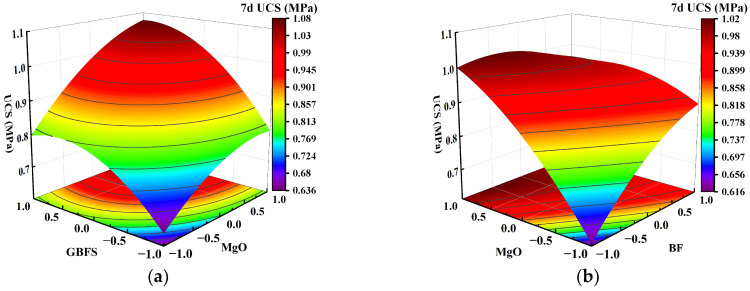
Effect of two-factor interactions on 7-day UCS. (**a**) GBFS content and MgO content; (**b**) MgO content and BF content.

**Figure 8:**
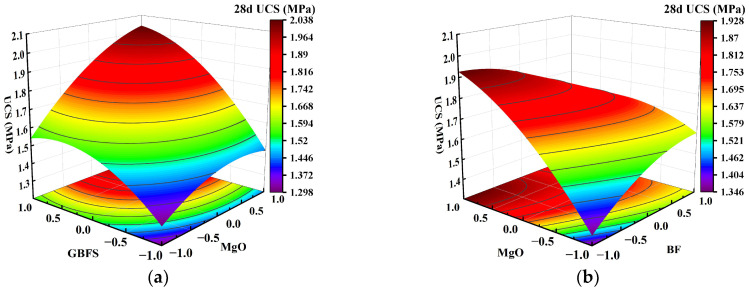
Effect of two-factor interactions on 28-day UCS. (**a**) GBFS content and MgO content; (**b**) MgO content and BF content.

**Figure 9:**
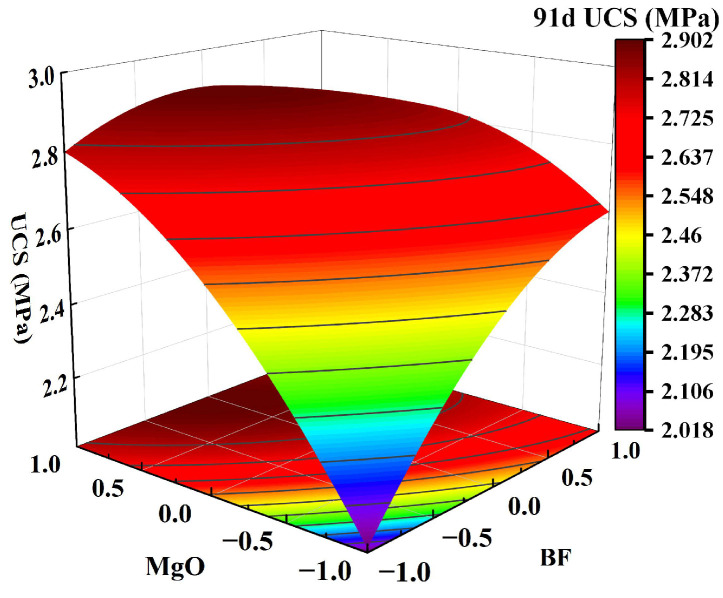
Effect of two-factor interactions on 91-day UCS: MgO content and BF content.

Using Design-Expert software, a response surface of the interaction between two factors on the UCS of solidified clay can be generated, as shown in Figure 7, Figure 8 and Figure 9. In the figure, red and blue colors indicate the larger UCS and the smaller UCS of the solidified clay, respectively. The steepness of the response surface indicates the more pronounced the interaction between the influencing factors. We selected 7 days, 28 days, and 91 days as the ages for factor interaction effect analysis of solidified clay to evaluate its early, standard, and long-term performance, respectively. As a transitional age, 14 days neither reflects the early performance as fast as 7 days, nor does it provides the standard comparability of 28 days; therefore, it is typically not utilized as the test age for factor interaction effects.

Figure 7a shows that as the GBFS content and MgO content increased, the response surface of the UCS of the solidified clay first increased and then decreased. The UCS of 1.5% MgO and 13.5% GBFS solidified clay was similar to the UCS of 4.5% MgO and 10.5% GBFS solidified clay. This observation suggests that the increase in UCS of solidified clay is inhibited in highly alkaline environments and at lower GBFS contents. Figure 7b shows that the UCS of the solidified clay increased continuously when the MgO content was increased from 1.5% to 4.5%. Similarly, the UCS of the solidified clay showed a continuous increase when the BF content was increased from 0.2% to 0.8%. However, the UCS of the solidified clay with 4.5% MgO and 0.2% BF was greater than that of the solidified clay with 1.5% MgO and 0.8% BF. This indicates that the role of MgO in improving the properties of clay is greater than that of BF. In Figure 8a, the trend of the UCS of the solidified clay shows a similar pattern to that in Figure 7a. Differences existed in that the UCS of 1.5% MgO and 13.5% GBFS solidified clay was greater than the UCS of 4.5% MgO and 10.5% GBFS solidified clay. In Figure 8b and Figure 9, the trend of UCS of solidified clay showed a similar pattern to that in Figure 7b. The above analysis shows that the combined action of GBFS, MgO, and BF can significantly increase the UCS of solidified clay.

### 3.5. Parameter Optimization and Experimental Validation

In the optimization process of the Design Expert software, the maximum UCS after 7, 28, and 91 days was used as the optimization objective. The prediction results indicated that the optimum mass percentages of GBFS, MgO, and BF were 13.39%, 4.5%, and 0.37%, respectively, at curing time for 7 days, resulting in a predicted UCS of 1.09 MPa. After 28 days, the optimum mass percentages were 13.5% for GBFS, 4.5% for MgO, and 0.2% for BF, corresponding to a predicted UCS of 2.1 MPa. After 91 days, the optimal mass percentages were 13.35% for GBFS, 4.47% for MgO, and 0.26% for BF, yielding a predicted UCS of 3.03 MPa. The test values of solidified clay were 0.93 MPa, 1.96 MPa, and 2.85 MPa after 7, 28, and 91 days, respectively, using the corresponding optimum mass percentage of stabilized clay. To assess the accuracy of the model, the absolute values of their relative deviation (ARD) are calculated using Equation (12).

(12) $$ARD=\frac{\left|V_{T}-V_{P}\right|}{V_{p}}\times 100\%$$

where *ARD* is the absolute value of relative deviation, *V_T_* is the UCS test value (MPa), and *V_P_* is the UCS prediction value (MPa).

The absolute value of relative deviation for solidified clay was 14.6%, 6.6%, and 5.9% for curing times of 7, 28, and 91 days, respectively. These results indicate that the quadratic regression model can fit the relationship between the mass percentage of curing materials and the UCS [47]. Therefore, in this study, a mass ratio of 13.35:4.47:0.26 for GBFS, MgO, and BF was selected for the curing materials. The curing materials used to solidify the clay were referred to as GMBF, where G stands for granulated blast furnace slag, M for magnesium oxide, and BF for basalt fiber.

### 3.6. UCS for GMBF Solidified Clay

#### 3.6.1. Compression Failure Mode and Stress–Strain Curve of Solidified Clay

**Figure 10:**
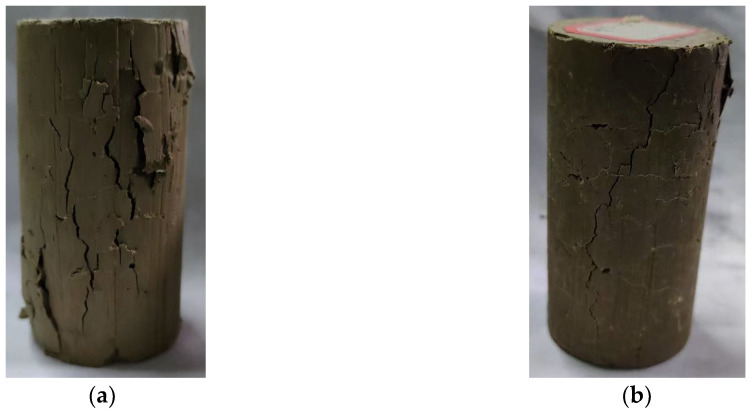
Typical compression failure mode of solidified clay. (**a**) GMBF solidified clay (**b**) OPC solidified clay.

The typical compressive failure modes of GMBF and OPC are shown in Figure 10. The GMBF solidified clay showed multiple short cracks, while the OPC solidified clay showed penetrating cracks extending from the top surface to the bottom of the sample. The difference in this destruction mode is mainly attributed to the presence or absence of BF. In GMBF solidified clay, the hydration reaction of GBFS and MgO generates gel substances that tightly bond BF with clay particles. The tensile strength of the blast furnace is relatively high (see Table 4). When GMBF solidified clay is compressed, friction occurs during the separation of BF from the clay or gel substances, which can resist part of the pressure applied to the sample [25,26]. Otherwise, BF is randomly distributed in the GMBF solidified clay, with BF acting as a bridge to disperse concentrated pressure, forming multiple ranges of common resistance [48]. This also indicates the presence of multiple short cracks on the surface of GMBF solidified clay. In OPC solidified clay, the hydration reaction of cement generates gel substances, which only combine with clay particles. In OPC solidified clay, the hydration reaction of cement generates gel substances that only bond with clay particles. When OPC solidified clay is compressed, under concentrated pressure, separation occurs between the particles. As the pressure increases, the cracks between the particles enlarge, eventually forming through cracks.

**Figure 11:**
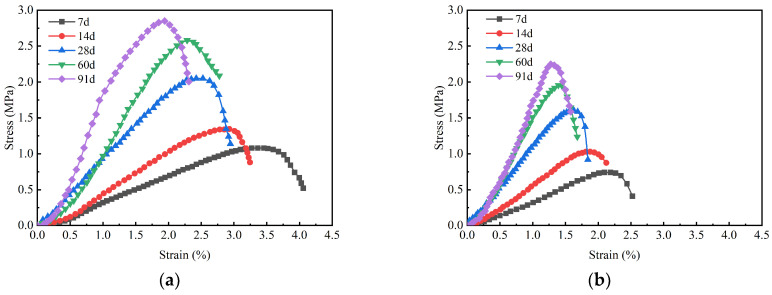
Stress–strain curve of solidified clay. (**a**) GMBF solidified clay (**b**) OPC solidified clay.

Figure 11 illustrates the stress–strain curves for GMBF solidified clay and OPC solidified clay. The strain corresponding to the peak axial stress of the sample is defined as the failure strain. The failure strain of GMBF solidified clay ranged from 1.94% to 3.36%, while the failure strain of OPC solidified clay ranged from 1.19% to 2.19%. The failure strain of the GMBF solidified clay was greater than those of the OPC solidified clay. From the perspective of failure strain, the presence of basalt fibers helps to improve the ductility of the solidified clay. The hydration reaction of GBFS with MgO produces gel substances. The soil particles are distributed around the BF, with the gel substances enhancing the bonding between the particles and the fibers. Together, the BF and the gel substances effectively confine the soil particles, promoting improved structural integrity [49,50].

The stress–strain curves of GMBF solidified clay and OPC solidified clay exhibited a similar trend prior to reaching the peak stress; specifically, the rate of stress increase in solidified clay gradually increased with the extension of curing time. For instance, after 7, 28, and 91 days, the failure strain of GMBF solidified clay was 3.36%, 2.53%, and 1.94%, respectively, while the failure strain of OPC solidified clay was 2.19%, 1.63%, and 1.19%, respectively. Compared to 7 days, the failure strain of GMBF solidified clay decreased by 24.7% and 42.3% after 28 days and 91 days, respectively, while the failure strain of OPC solidified clay decreased by 25.6% and 45.7% after 28 days and 91 days, respectively. This observation indicates that the failure strain tends to decrease as compressive strength increases, a finding that generally aligns with the experimental results reported by other researchers [37,51,52].

#### 3.6.2. Changing Pattern of UCS in Solidified Clay with Curing Time

**Figure 12:**
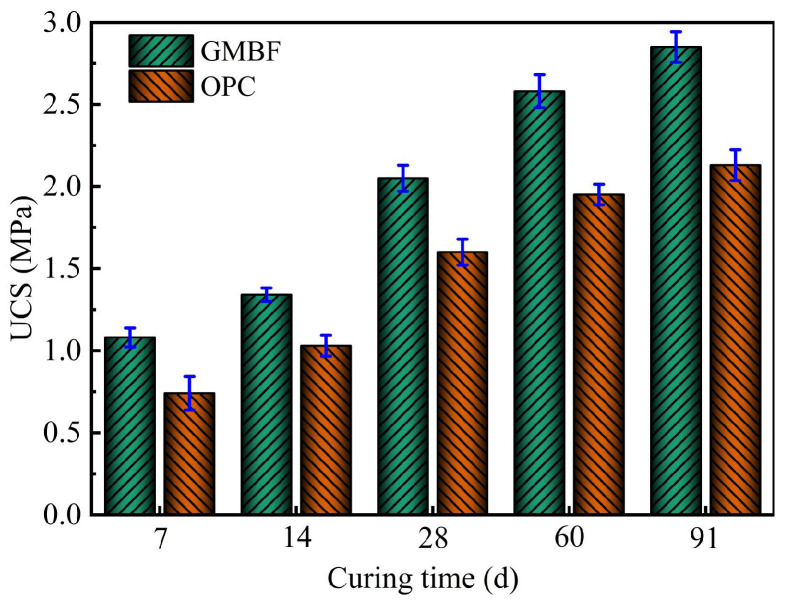
Relationship between solidified clay and curing time.

Figure 12 illustrates the average UCS of GMBF solidified clay and its standard deviation. The average UCS of OPC solidified clay with the same curing materials dosage is also plotted in the figure for comparison. As shown in Figure 12, the UCS of the GMBF solidified clay sample increased with curing time and was consistently higher than that of the OPC solidified clay over the curing time from 7 to 91 days. Specifically, the UCS of GMBF solidified clay increased by 45.9%, 28.1%, and 33.8% over OPC solidified clay after 7, 28, and 91 days, respectively.

The reasons for the high UCS of GMBF solidified clay may include the following [25,26,53,54]: ① The silicates in the GBFS consist of silicate tetrahedra (SiO_4_) and metal cations Ca^2+^. When GBFS, MgO, and BF are mixed with clay, MgO dissolves in the moist clay and releases hydroxide (OH^−^), thereby increasing the alkalinity of the pore solution, as shown in Equation (13). Under alkaline conditions, the silicates in the GBFS (such as CaSiO_3_) react with water to form C-S-H gel, as shown in Equation (14). ② The contents of SiO_2_ and CaO in GBFS were 35% and 40.1%, respectively, while the content of SiO_2_ in clay was 72.1% (see Table 3). When GBFS is mixed with clay in an alkaline environment, C-S-H gel is formed during the hydration process. In other words, CaO dissolves in the moist clay to form Ca(OH)_2_, as illustrated in Equation (15), and Ca(OH)_2_ reacts with reactive SiO_2_ to form C-S-H gel products, as exhibited in Equation (16). ③ MgO hydrates to form Mg(OH)_2_. In an alkaline environment, Al_2_O_3_ in the GBFS dissolves to form Al^3+^. Mg^2+^ and Al^3+^ combine with CO_3_^2−^ or OH^ࢤ^ to form hydrotalcite (Ht, Mg_6_Al_2_(OH)_16_CO_3_·4H_2_O), as shown in Equation (17). Ht has a certain degree of expansion, which can fill the pores between clay particles, thereby enhancing the strength of the clay. ④ A good interface bond is formed between BF and gel substances, while BF can effectively transmit stress and alleviate local stress concentration. These hydration reactions and their products will be further validated in microscopic analysis. Overall, the gel substances formed by GBFS and MgO create a composite structure with BF, fully utilizing their respective advantages to achieve efficient solidification of clay.

The main mineral components of OPC were C_3_S and C₂S (see Figure 3d). In OPC solidified clay, OPC reacts with water to form C-S-H gel and Ca(OH)_2_. In GMBF solidified clay, an interface bond occurs between the BF and the clay or substances, whereas OPC solidified clay does not exhibit this interface bond. This absence is one of the reasons why the unconfined compressive strength (UCS) of OPC solidified clay is lower than that of GMBF solidified clay.

(13) $$MgO+H_{2}O\to Mg{\left(OH\right)}_{2}\to Mg^{2+}+OH^{-}$$

(14) $$CaSiO_{3}+H_{2}O\to C-S-H+Ca{\left(OH\right)}_{2}$$

(15) $$CaO+H_{2}O\to Ca{\left(OH\right)}_{2}\to Ca^{2+}+OH^{-}$$

(16) $$Ca{\left(OH\right)}_{2}+SiO_{2}+H_{2}O\to C-S-H$$

(17) $$Mg^{2+}+Al^{3+}+OH^{-}+C{O_{3}}^{2-}\to Ht$$

**Figure 13:**
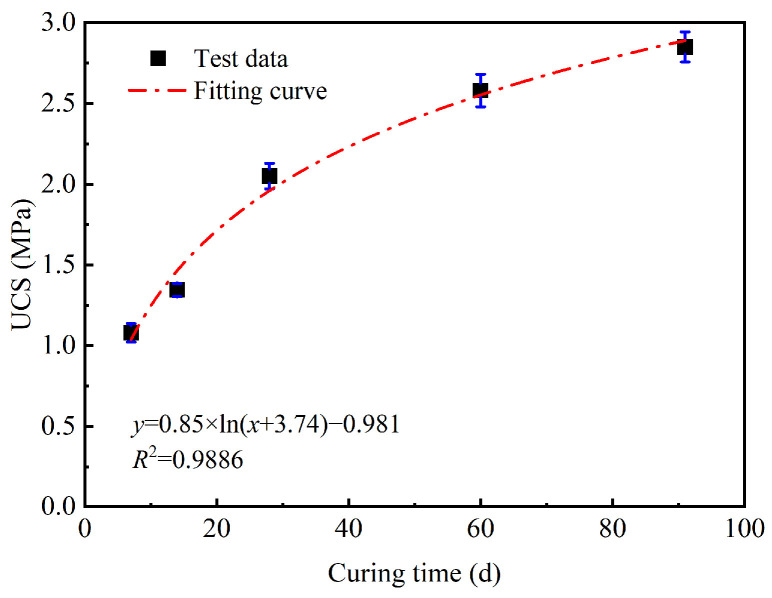
Curve of UCS of GMBF solidified clay with curing time.

The UCS of GMBF solidified clay over the curing times from 7 to 91 days was fitted using a logarithmic function, with the fitted curves illustrated in Figure 13. The correlation coefficient, *R*^2^, of this curve was 0.9886, indicating a strong correlation. However, this equation could only predict the UCS at 7 days and above and could not estimate the UCS at earlier times. For curing materials used in roadbed soils, later UCS is a critical factor [3]. Therefore, the curve can be used to predict the relationship between the UCS of solidified clay and curing time.

### 3.7. Water Stability for Solidified Clay

**Figure 14:**
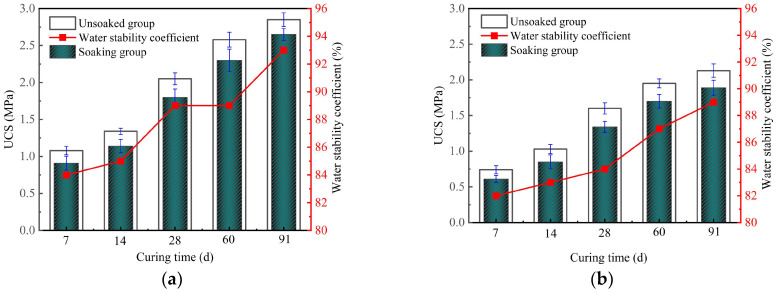
Effect of water soaking on UCS of solidified clay. (**a**) GMBF solidified clay (**b**) OPC solidified clay.

The water stability of GMBF solidified clay and OPC solidified clay is shown in Figure 14. As can be seen in Figure 14, the UCS of the solidified clay after undergoing 24 h of water soaking was lower than the UCS of the solidified clay when it was not soaked. The water stability coefficients of the GMBF solidified clay and OPC solidified clay exceeded 80% at curing times of 7, 14, 28, 60, and 91 days, thus meeting the practical requirements for water stability of solidified clay [6,43]. The water stability coefficients of GMBF solidified clay were consistently greater than those of OPC solidified clay. Specifically, the water stability coefficients of GMBF solidified clay were 84%, 89%, and 93% after 7, 28, and 91 days, respectively. The water stability coefficients of GMBF solidified clay were 2.44%, 5.95%, and 4.49% higher than those of OPC solidified clay at the same time. The confinement effect of BF on the particles enhances the water stability of GMBF solidified clay compared to that of OPC solidified clay. The water stability coefficient of solidified clay exceeds 80%, mainly for the following reasons [55]. ① The cementing effect of gel substances: The hydration products of GBFS and MgO consist of C-S-H gel, while those of OPC also yield C-S-H gel. These gel substances provide skeletal support for the solidified clay by cementing particles and filling the pores, thereby mitigating the adverse effects of free water on the particles. ② Ion exchange between particles: As we all know, Na^+^ and K^+^ were present in the clay. The residual Ca^2+^ in the GBFS or OPC is more likely to undergo ion exchange with Na^+^ and K^+^ in the clay, resulting in a decrease in the thickness of the double electric layer, which subsequently enhances the gravitational force between the particles, enabling the solidified clay to sustain a high UCS.

The effect of water immersion on the UCS of solidified clay is primarily attributed to the following factors [56]: ① As can be seen from Figure 4a, particles with a size of less than 2 µm in the clay are called clay, which account for 21.9% of the total number of particles. When clay particles absorb water, their volume changes and expands. ② From Figure 3a, it can be seen that the mineral composition of clay contains montmorillonite, which undergoes volume expansion when exposed to water. In summary, the cementation between particles of solidified clay is weakened under water-immersed conditions, resulting in a decrease in overall structural strength. This phenomenon is due to physical changes in the water-sensitive components of the clay in the presence of water. Therefore, in order to maintain the higher mechanical properties of solidified clay, its practical application should have avoided prolonged soaking in water.

### 3.8. Freeze–Thaw Cycle for Solidified Clay

#### 3.8.1. Moisture Content, Density, and Mass Loss Rate of Solidified Clay

**Figure 15:**
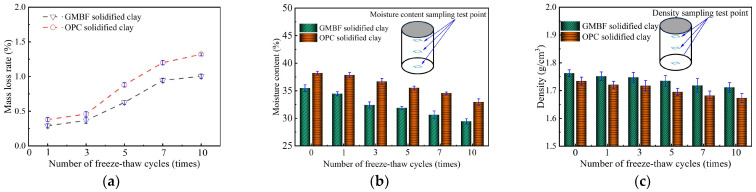
Freeze–thaw cycle of solidified clay. (**a**) Mass loss rate of solidified clay; (**b**) Moisture content of solidified clay; (**c**) Density of solidified clay.

Table 10 lists the physical indicators of GMBF solidified clay and OPC solidified clay under a standard curing time of 91 days. The relationship curves between the moisture content, density, and mass loss rate of solidified clay and the number of freeze–thaw cycles are shown in Figure 15.

As can be seen in Figure 15a, the mass loss rate of GMBF solidified clay particles was always lower than that of OPC solidified clay particles during the freeze–thaw cycle. Moreover, the random distribution of BF in the GMBF solidified clay served to constrain the particles, thereby reducing the shedding of particles from the specimen surface during the freeze–thaw cycle [57]. The freeze–thaw cycle test employs the dry-freezing method. Specifically, the samples were thawed at a room temperature of 20 °C after freezing. The loss of moisture mass in the samples primarily resulted from changes in water location during freezing and natural evaporation from the surface during thawing at room temperature. The moisture content of the solidified clay exhibited a decreasing trend as the number of freeze–thaw cycles increased. As indicated in Table 10, the moisture content of OPC solidified clay was higher than that of GMBF solidified clay. The moisture content of OPC solidified clay consistently exceeded that of GMBF solidified clay throughout the freeze–thaw cycles, as shown in Figure 15b. Figure 15c illustrates that the densities of both GMBF solidified clay and OPC solidified clay decreased with an increasing number of freeze–thaw cycles. Specifically, compared to the initial state (zero freeze–thaw cycles), the densities of GMBF solidified clay and OPC solidified clay decreased by 2.9% and 3.5%, respectively, after ten freeze–thaw cycles. This observation indicates that freeze–thaw cycles significantly affect the compactness of solidified clay, which can be reflected by the change in its UCS.

#### 3.8.2. UCS of Solidified Clay

**Figure 16:**
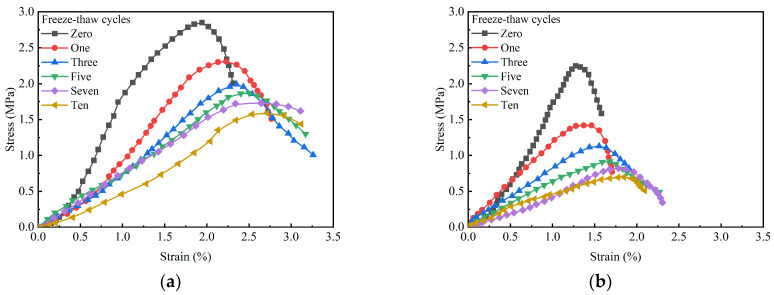
Stress–strain curves of solidified clay subjected to varying numbers of freeze–thaw cycles. (**a**) GBFS solidified clay; (**b**) OPC solidified clay.

Figure 16 illustrates the stress–strain curves of GMBF solidified clay and OPC solidified clay after different numbers of freeze–thaw cycles. With the increase in the number of freeze–thaw cycles, the peak stress of GMBF solidified clay and OPC solidified clay both decreased, while the peak strain increased. The peak strains at zero freeze–thaw cycles were 1.92% and 1.19% for GMBF solidified clay and OPC solidified clay, respectively. After ten freeze–thaw cycles, the peak strains of GMBF solidified clay and OPC solidified clay were 2.71% and 1.85%, respectively. At the same number of freeze–thaw cycles, the peak strain of OPC solidified clay was smaller than that of GMBF solidified clay. Analysis of the peak strain values reveals that the increase in peak strain for OPC solidified clay without BF was relatively modest, whereas the increase in peak strain for GMBF solidified clay containing BF was relatively greater. After the peak strain, the stresses in the GMBF solidified clay decreased relatively slowly, whereas the stresses in the OPC solidified clay decreased relatively quickly. Bonding between solidified clay particles weakens with the number of freeze–thaw cycles. The friction between BF and particles or gel substances in GMBF solidified clay can reduce the adverse effects of freeze–thaw cycles, manifested as an increase in strain with the number of freeze–thaw cycles and a decrease in stress with the number of freeze–thaw cycles [57]. BF also played an important role in the freeze–thaw cycle of GMBF solidified clay.

**Figure 17:**
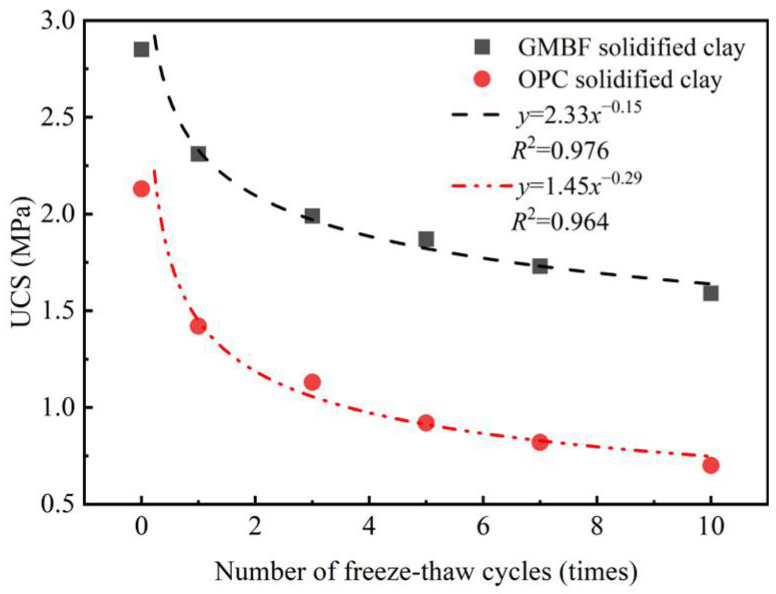
Relationship between the UCS of solidified clay and the number of freeze–thaw cycles.

Figure 17 illustrates the relationship between the UCS of solidified clay and the number of freeze–thaw cycles. The UCS of both GMBF solidified clay and OPC solidified clay decreased as the number of freeze–thaw cycles increased. After one freeze–thaw cycle, the UCS values of GMBF solidified clay and OPC solidified clay were 2.31 MPa and 1.42 MPa, respectively, reflecting decreases of 33% and 19% compared to their values at zero freeze–thaw cycles. In the unfrozen state, the moisture content of GMBF solidified clay and OPC solidified clay was 35.46% and 38.21%, respectively (see Figure 15b). During the freezing process, the moisture in the solidified clay was converted from liquid to ice. When the pore volume in the solidified clay cannot accommodate the volume of the ice mass, the position of the particles is displaced, breaking the connections between the particles and generating new cracks [58]. After ten freeze–thaw cycles, the UCS values of GMBF solidified clay and OPC solidified clay were 1.59 MPa and 0.7 MPa, respectively, which decreased by 67% and 44% compared to zero freeze–thaw cycles. The cracks that have formed in the solidified clay do not return to the state they were in before the freeze–thaw cycle due to the melting of the ice. After the ice melts, the distribution of water shifts. Existing cracks in solidified clay continue to propagate during freeze–thaw cycles due to changes in water distribution [59,60]. Repeated freeze–thaw cycles contribute to the structural deterioration of the solidified clay, subsequently leading to a reduction in its macroscopic mechanical properties.

The power function was used to establish the relationship between the UCS of the solidified clay and the number of freeze–thaw cycles, and the fitting results are shown in Figure 17. The coefficients of determination for GMBF solidified clay and OPC solidified clay were 0.976 and 0.964, respectively, which were both greater than 0.95. Numerical analysis indicates that a power function more accurately describes the relationship between the UCS of solidified clay and the number of freeze–thaw cycles.

## 4. Microscopic Test Results for Solidified Clay

### 4.1. X-Ray Diffraction

**Figure 18:**
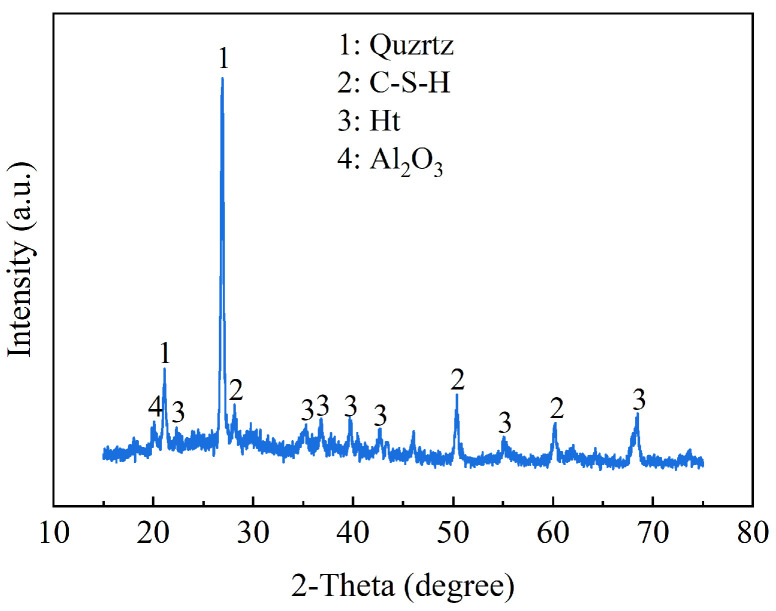
XRD patterns of solidified clay.

X-ray diffraction tests were used to determine the hydration products of solidified clay, as shown in Figure 18. XRD revealed the presence of C-S-H phase, hydrotalcite (Ht) phase, alumina (Al_2_O_3_), and quartz in GMBF solidified clay. Active MgO undergoes a hydration reaction interacting with water, resulting in the production of hydroxide ions (OH^−^) and magnesium ions (Mg^2+^). The OH^-^ subsequently enhances the alkalinity of the matrix environment. The GBFS predominantly exists in a glassy structure, primarily consisting of silicon oxygen tetrahedra and aluminum oxygen tetrahedra [61]. The silica–oxygen tetrahedron in the GBFS exhibits a low degree of polymerization and can dissociate in the presence of OH^-^, leading to the formation of C-S-H gel, as evidenced by the diffraction peak observed at 2*θ* = 28.26° [21,62,63]. The Mg^2+^ from MgO and the Al^3+^ present in the GBFS or SC participate in a hydration reaction, resulting in the formation of the Ht phase, as indicated by the diffraction peak observed at 2*θ* = 43.4° [64].

### 4.2. Scanning Electron Microscopy

**Figure 19:**
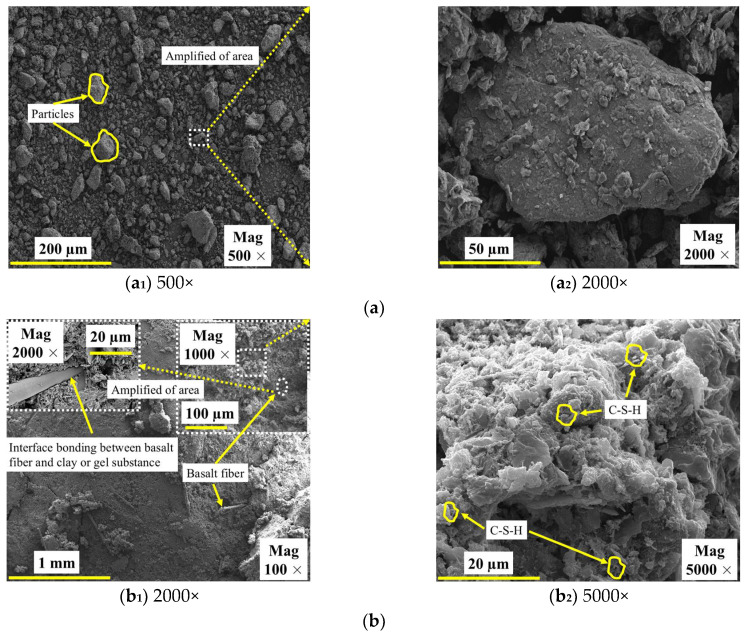
SEM images of SC and solidified clay after 91 days of curing. (**a**) SC; (**b**) GMBF solidified clay.

The microscopic morphological characteristics of SC and GMBF solidified clay are illustrated in Figure 19. The SC consists of particles of varying sizes, with a notable lack of effective interconnections among them upon mixing SC with GMBF to form GMBF solidified clay. After 91 days of curing, microscopic images of the GMBF solidified clay reveal the formation of effective bonds between the particles, which can be attributed to the presence of hydration products [65]. Compared to untreated clay, GMBF solidified clay demonstrates a denser structure with significantly reduced porosity. The particles encapsulate the BF because the hydration products are distributed on the surface of the fibers, resulting in close contact between the particles and the BF, which is facilitated by the bonding properties of these hydration products [27,66]. On the one hand, this interface bonding effect enhanced the interaction between the particles and the BF, confirming that the use of BF can improve the UCS of solidified clay. On the other hand, the binding effect of the hydration products leads to a typical agglomerated distribution of particles within the solidified clay, while the BF can connect these agglomerates, enhancing the linkage between the agglomerates. Specifically, when a force is applied to the specimen, the stress is transferred through the frictional interaction between the deformation of the BF and the particles or gel substances [25,48].

## 5. Discussion

### 5.1. Mechanism of Action of Curing Materials on Clay

First, based on the central composite design in the response surface, the optimum mass ratio of GBFS, MgO, and BF was obtained as 13.35:4.47:0.26, and the resulting composition of the curing materials was named GMBF. An equal amount of OPC was taken and formed with SC to form OPC solidified clay. Then, through the UCS test, water stability test, and freeze–thaw cycle test, it was evident that the mechanical properties and durability of GMBF solidified clay were superior to those of OPC solidified clay (see Figure 12, Figure 14 and Figure 17). From the stress–strain curves (see Figure 11), the peak strain of the GMBF solidified clay was greater than the peak strain of the OPC solidified clay. Under freeze–thaw cycles, GMBF solidified clay exhibited a relatively slow rate of stress reduction following peak stress, whereas OPC solidified clay demonstrated a comparatively rapid rate of stress reduction after peak stress (see Figure 19). Furthermore, the GBFS exhibited a low degree of polymerization and can dissociate in the presence of OH^−^, leading to the formation of C-S-H gel [20,21]. Finally, the hydration products of GBFS and MgO make the clay tightly bonded to the basalt fibers (see Figure 16). Interface friction effects occur between basalt fibers and particles or hydration products, and basalt fibers form spatial constraints on particles. The contribution of basalt fiber to the properties of solidified clay is mainly reflected in the following aspects [25,26]:

(I) Interface bond: Basalt fibers with rough surfaces were tightly bonded to the particles or hydration products, and this tight association was continually enhanced by the development of the hydration products. When subjected to compression, the bonding forces generated by the hydration products work in conjunction with the friction forces generated by the basalt fibers to achieve a high load-bearing capacity.

(II) Bridging effect: The bridging effect exerted by the right amount of basalt fibers improves the curing integrity. When the solidified clay was subjected to compression, the basalt fibers acted as a bridge to disperse the concentrated pressure, creating multiple ranges of common resistance. After the solidified clay reached its peak stress, the basalt fibers and hydration products gradually separated under external pressure but still maintained resistance to the external pressure, which led to the formation of multiple cracks (see Figure 10). Moreover, the stress in the solidified clay decreased slowly with increasing strain.

### 5.2. Potential Applications and Further Research of Solidified Clay

Zhang et al. mixed O-QGS with clay to form a mixture, made prefabricated piles by pressurization, and installed the prefabricated O-QGS solidified soil piles into the ground using a hydraulic static pile driver to reinforce the soft ground [6]. Pallav et al. stabilized peat soils using oil shale ash and pozzolanic additives, and test results from experimental sections indicated that the compressive strength of the treated peat soils was sufficient for the construction of roads with medium bearing capacity [7].

It can be seen that the reuse of clay can be achieved by adding curing materials to clay with poor mechanical properties. This also suggests that GMBF solidified clay can be utilized for pavement subgrade, as this indoor experimental study showed that GMBF solidified clay not only meets the strength requirements (Figure 12) but also exhibits a high failure strain (Figure 11), which is hypothesized to reduce the risk of pavement subgrade cracking. For deeper clay layers, deep mixing equipment may be considered to mix the curing material with the clay to form deep mixing piles. However, this method places greater demands on the equipment and necessitates ensuring the homogeneity of the mixture of the curing material with the clay. Additionally, MgO is utilized in this study as an alkaline activator for GBFS, which is less alkaline than lime and calcium carbide slag and has less impact on the surrounding environment. Although the cost of MgO is high when applied over large areas, the overall construction costs can be reduced by using waste slag. The combination of GBFS, MgO, and BF was effective on clay in a certain foundation pit in Hangzhou, China; however, its applicability to soft clay in other regions will be the focus of future research.

## 6. Conclusions

In this study, a curing material named GMBF was developed based on CCD in RSM and mixed with clay to form GMBF solidified clay. Through a series of laboratory tests, the mechanical properties, durability, and micro-morphology of the GMBF solidified clay were obtained, and the contribution of basalt fibers in the solidified clay was further clarified. The main conclusions are as follows:

(1) A quadratic regression model of UCS was developed for 7, 28, and 91 days, and analysis of variance and experimental validation showed that the model was well fitted and accurately predicted to obtain the optimal mix ratio. Based on the parameter optimization, the optimum mass ratio of GBFS, MgO, and BF in GMBF was 13.35:4.47:0.26.

(2) The UCS values of GMBF solidified clay after 7 days, 28 days, and 91 days were 1.08 MPa, 2.05 MPa, and 2.85 MPa, respectively. At curing times of 7 days and 91 days, the UCS of GMBF solidified clay increased by 45.9% and 33.8% compared to OPC solidified clay, respectively.

(3) The water stability coefficient of GMBF solidified clay after 7 days, 28 days, and 91 days was 84%, 89%, and 93%, respectively. The water stability coefficient of GMBF solidified clay was improved by 2.44%, 5.95%, and 4.49% compared to that of OPC solidified clay. The GMBF solidified clay experienced ten freeze–thaw cycles, which resulted in a decrease of less than 50% compared to zero freeze–thaw cycles, with a specific decrease of 44.2%. In contrast, the decline of OPC solidified clay exceeded 50%.

(4) The hydration products of GMBF solidified clay primarily consisted of C-S-H gel and Ht. The contribution of basalt fibers to GMBF solidified clay was explained by the interaction of the fibers with particles or hydration products, including bonding and bridging effects. This also led to good overall properties of GMBF solidified clay, resulting in better mechanical and durability properties.

## Figures and Tables

**Table 1:** Physical indexes of SC.

Value	Physical and Mechanical Index							
	Natural Density (g/cm^3^)	Specific Gravity	Liquid Limit (%)	Plastic Limit (%)	Plasticity Index	Void Ratio	Maximum Dry Density (g/cm^3^)	Optimum Water Content (%)
Average value	1.68	2.68	42.5	22.6	19.9	1.47	1.56	22
Standard deviation	0.00733	0.00311	0.35637	0.42071	/	/	0.00235	0.15811

**Table 2:** Physical properties of OPC.

Value	Fineness (%)	Setting Time (min)		Loss on Ignition (%)	Bulk Density (g/cm^3^)	Density (g/cm^3^)	Specific Surface Area (m^2^/kg)	Unconfined Compressive Strength (MPa)	
		Initial	Final					3 d	28 d
Average value	3.1	192	372	4.5	1.31	3.07	351	24.1	48.8
Standard deviation	0.15811	3.83406	5.70088	0.40373	0.0142	0.0255	2.60768	0.68337	1.17132

**Table 3:** Major chemical compositions (wt.%) of SC, GBFS, MgO, and OPC.

Composition Content	SiO_2_	CaO	Al_2_O_3_	Fe_2_O_3_	SO_3_	K_2_O	MgO	Others
SC	72.1	0.43	17.5	4.2	2.9	1.4	0.9	0.57
GBFS	35	40.1	14.1	5.29	2.51	0.55	1.1	1.35
MgO	/	0.55	1.12	0.12	0.11	0.01	95.3	2.79
OPC	18.9	64.55	4.95	2.39	2.65	1.26	0.88	4.42

**Table 4:** Performance parameters of BF.

Performance Parameters	Monofilament Diameter (μm)	Tensile Strength (MPa)	Ultimate Elongation (%)	Fiber Type	Elastic Modulus (GPa)	Acid and Alkali Resistance (%)	Density (g/cm^3^)
Value	16.5	3000~4500	2.0~3.15	Monofilament bundle	95–105	≥99	2.61

**Table 5:** Independent variables and coding levels for central composite design.

Independent Variable	Code	Levels of Central Composite Design				
		Minimum	Low	Middle	High	Maximum
GBFS	*X*_1_	−1.682 (9.48)	−1 (10.5)	0 (12)	1 (13.5)	1.682 (14.52)
MgO	*X*_2_	−1.682 (0.48)	−1 (1.5)	0 (3)	1 (4.5)	1.682 (5.52)
BF	*X*_3_	−1.682 (0)	−1 (0.2)	0 (0.5)	1 (0.8)	1.682 (1.00454)

**Table 6:** Comparative test scheme.

Type of Solidified Clay	Type of Test	Curing Time (day)	Number of Freeze–Thaw Cycles (Times)
GMBF solidified clay and OPC solidified clay	UCS	7, 14, 28, 60, 91	/
	Water stability	7(6), 14(13), 28(27), 60(59), 91(90)	/
	Freeze–thaw cycle	91	1, 3, 5, 7, 10
GMBF solidified clay	SEM and XRD	91	/

**Table 7:** Relationship between the content of curing materials and the UCS of solidified clay.

Group	Mass of GBFS, MgO, and BF as a Percentage of Dry Clay Mass (%)			UCS (MPa)		
	GBFS	MgO	BF	7d	28d	91d
S-A1	13.5	1.5	0	0.696	1.503	2.129
S-A2	12	3	0	0.875	1.586	2.239
S-A3	10.5	4.5	0	0.778	1.419	1.949
S-A4	9	6	0	0.569	1.143	1.589
S-B1	12	3	0.2	0.939	1.742	2.257
S-B2	12	3	0.4	0.969	1.895	2.679
S-B3	12	3	0.6	1.019	2.189	2.766
S-B4	12	3	0.8	0.969	2.204	2.697

**Table 8:** Arrangement and results of central composite design tests.

Group	Independent Variable (%)			Response Value (MPa)		
	*X*_1_	*X*_2_	*X*_3_	*Y*_7*d*_	*Y*_28*d*_	*Y*_91*d*_
1	−1 (10.5)	−1 (1.5)	−1 (0.2)	0.501	1.07	1.71
2	1 (13.5)	−1 (1.5)	−1 (0.2)	0.632	1.35	2.1
3	−1 (10.5)	1 (4.5)	−1 (0.2)	0.794	1.38	2.19
4	1 (13.5v	1 (4.5)	−1 (0.2)	1.098	2.12	3.01
5	−1 (10.5)	−1 (1.5)	1 (0.8)	0.749	1.43	2.38
6	1 (13.5)	−1 (1.5)	1 (0.8)	0.915	1.7	2.66
7	−1 (10.5)	1 (4.5)	1 (0.8)	0.768	1.31	2.33
8	1 (13.5)	1 (4.5)	1 (0.8)	0.999	1.82	2.84
9	−1.682 (9.48)	0 (3)	0 (0.5)	0.505	1.15	1.72
10	1.682 (14.52)	0 (3)	0 (0.5)	0.912	1.77	2.66
11	0 (12v	−1.682 (0.48)	0 (0.5)	0.589	1.24	1.92
12	0 (12)	1.682 (5.52)	0 (0.5)	0.999	1.88	2.85
13	0 (12)	0 (3)	−1.682 (0)	0.724	1.55	2.22
14	0 (12)	0 (3)	1.682 (1.00454)	0.892	1.67	2.61
15	0 (12)	0 (3)	0 (0.5)	0.959	1.77	2.76
16	0 (12)	0 (3)	0 (0.5)	0.914	1.7	2.66
17	0 (12)	0 (3)	0 (0.5)	0.945	1.75	2.93
18	0 (12)	0 (3)	0 (0.5)	0.954	1.76	2.74
19	0 (12)	0 (3)	0 (0.5)	0.974	1.79	2.8
20	0 (12)	0 (3)	0 (0.5)	0.991	1.93	2.94

**Table 9:** ANOVA and significance test results of regression model coefficients for solidified clay after 7 days, 28 days, and 91 days.

	7 Days			28 Days			91 Days		
Source	Sum of Squares	F-Value	*p*-Value	Sum of Squares	F-Value	*p*-Value	Sum of Squares	F-Value	*p*-Value
Model	0.56	44.46	<0.0001	1.46	31.06	<0.0001	3.0	25.45	<0.0001
*X*_1_	0.17	119.6	<0.0001	0.59	113.36	<0.0001	0.94	71.76	<0.0001
*X*_2_	0.18	125.2	<0.0001	0.34	65.23	<0.0001	0.7	53.23	<0.0001
*X*_3_	0.035	24.88	0.0005	0.022	4.2	0.0675	0.25	19.47	0.0013
*X*_1_ *X*_2_	0.00708	5.03	0.0488	0.061	11.73	0.0065	0.054	4.16	0.0687
*X*_1_ *X*_3_	0.00018	0.13	0.7277	0.0072	1.38	0.2674	0.022	1.69	0.2234
*X*_2_ *X*_3_	0.054	38.21	0.0001	0.15	27.93	0.0004	0.2	15.17	0.003
*X*_1_^2^	0.086	61.17	<0.0001	0.19	36.85	0.0001	0.55	42.22	<0.0001
*X*_2_^2^	0.032	22.68	0.0008	0.093	17.74	0.0018	0.23	17.72	0.0018
*X*_3_^2^	0.026	18.26	0.0016	0.057	10.86	0.0081	0.2	15.0	0.0031
Residual	0.014	/	/	0.052	/	/	0.13	/	/
Lack of fit	0.011	3.08	0.1209	0.022	0.72	0.6359	0.07	1.14	0.6359
Pure error	0.00345	/	/	0.03	/	/	0.061	/	/
Cor total	0.058	/	/	1.51	/	/	3.13	/	/
Standard deviation	0.038			0.072			0.11		
C.V. (%)	4.46			4.5			4.57		
R^2^	R^2^ = 0.9756, Adj R^2^ = 0.9537, and Pred R^2^ = 0.8482			R^2^ = 0.9655, Adj R^2^ = 0.9344, and Pred R^2^ = 0.8547			R^2^ = 0.9582, Adj R^2^ = 0.9205, and Pred R^2^ = 0.8002		
Adeq precision	23.473			20.79			17.26		

**Table 10:** Parameters for solidified clay in standard curing for 91 days.

Sample	Value	Parameter			
		Moisture Content (%)	Volume (cm^3^)	Density (g/cm^3^)	Mass (g)
GMBF solidified clay	Average value	35.46	96.06	1.76	166.59
	Standard deviation	0.59551	/	0.01172	1.91105
OPC solidified clay	Average value	38.21	96.06	1.73	169.34
	Standard deviation	0.33633	/	0.01421	1.05324

## Data Availability

Data are contained within the article.
